# RETRACTED: Patano et al. Mandibular Crowding: Diagnosis and Management—A Scoping Review. *J. Pers. Med.* 2023, *13*, 774

**DOI:** 10.3390/jpm16110542

**Published:** 2026-10-23

**Authors:** Assunta Patano, Giuseppina Malcangi, Alessio Danilo Inchingolo, Grazia Garofoli, Nicole De Leonardis, Daniela Azzollini, Giulia Latini, Antonio Mancini, Vincenzo Carpentiere, Claudia Laudadio, Francesco Inchingolo, Silvia D’Agostino, Daniela Di Venere, Gianluca Martino Tartaglia, Marco Dolci, Gianna Dipalma, Angelo Michele Inchingolo

**Affiliations:** 1 Department of Interdisciplinary Medicine, University of Bari “Aldo Moro”, 70124 Bari, Italy; 2 Department of Medical, Oral and Biotechnological Sciences, University G. D’Annunzio, 66100 Chieti, Italy; 3 Department of Biomedical, Surgical and Dental Sciences, School of Dentistry, University of Milan, 20100 Milan, Italy; 4 UOC Maxillo-Facial Surgery and Dentistry, Fondazione IRCCS Cà Granda, Ospedale Maggiore Policlinico, 20100 Milan, Italy

The journal retracts the review article titled “Mandibular Crowding: Diagnosis and Management—A Scoping Review” [1], cited above.

Following publication of a correction [1], additional concerns were raised regarding the accuracy of cited sources and the methodology used in this systematic review.

In accordance with the journal’s standard procedures, the Editorial Office and Editorial Board conducted an investigation that identified major methodological flaws, including inconsistent and non-standard descriptions of study designs, inappropriate risk-of-bias assessment reporting, misclassification of unrelated studies, and other methodological concerns.

Consequently, the Editorial Board has lost confidence in the reliability of the review and has decided to retract this publication in accordance with MDPI’s retraction policy.

This retraction was approved by the Editor-in-Chief of the *Journal of Personalized Medicine*.

The authors have been informed of this retraction and have indicated their disagreement with this decision.
